# Supplementary material for: Predictors of mortality and loss to follow-up among drug resistant tuberculosis patients in Oromia Hospitals, Ethiopia: A retrospective follow-up study
Source: PLoS One. 2021 May 6;16(5):e0250804. doi: 10.1371/journal.pone.0250804 (PMC8101723; doi:10.1371/journal.pone.0250804)
Supplement: Attachment — Submitted filename: Responses to reviewers 2.docx [file pone.0250804.s002.docx]

**Responses for the comments from reviewers**

We are very grateful to the editor and reviewers for their valuable comments and we believe that the revised manuscript is much stronger as a result of their feedback.

**Dear Editor and reviewers:**

Thank you for the opportunity to revise our manuscript “Predictors of Mortality and Loss to Follow-up among Drug Resistant Tuberculosis Patients in Oromia Hospitals, Ethiopia: A Retrospective Follow-up Study” (PONE-D-21-04578R1) for publication by PLOS ONE.

We have addressed the Editors’ and reviewers’ comments in our responses and incorporated related changes into the manuscript. For convenience, the reviewers’ comments are in **black** and our responses are in **blue.**

The primary changes made to the study include: Editing of the manuscript to improve clarity and consistency

1. According to Table 1, the study included data from minors (age <18). In your Methods section, please ensure you have also stated whether you obtained consent from parents or guardians of the minors included in the study or whether the research ethics committee or IRB specifically waived the need for parental consent.

**Responses: Thank very much for flagging this: Under age 18 study participants were included due to editorial problem. They are under age category of 19 to 24.**

2. Please amend the title either on the online submission form or in your manuscript so that they are identical.

**Responses: We made correction**

3. Please ensure that you refer to Figure 2 and Figure 3 in your text as, if accepted, production will need this reference to link the reader to the figures.

**Responses: We made correction**

4. Please upload a copy of Supporting Information Table S1 - S4 which you refer to in your text.

**Responses: We made correction**

5. Thank you for updating your data availability statement. You note that your data are available within the Supporting Information files, but no such files have been included with your submission. At this time we ask that you please upload your minimal data set as a Supporting Information file, or to a public repository such as Figshare or Dryad. Please also ensure that when you upload your file you include separate captions for your supplementary files at the end of your manuscript.

**Responses: We have uploaded as supplementary file**
